# Cytokine storm and COVID-19: a chronicle of pro-inflammatory cytokines

**DOI:** 10.1098/rsob.200160

**Published:** 2020-09-23

**Authors:** Antonella Fara, Zan Mitrev, Rodney Alexander Rosalia, Bakri M. Assas

**Affiliations:** 1 Independent researcher, unaffiliated; 2 Department of Clinical Research, Zan Mitrev Clinic, St. Bledski Dogovor 8, 1000 Skopje, The Republic of North Macedonia; 3 Faculty of Applied Medical Sciences, Department of Medical Laboratory Technology, Immunology group, King Abdul Aziz University, Jeddah, Saudi Arabia

**Keywords:** COVID-19, IL-6, TNF-α, IFN-γ, SARS, coronavirus

## Abstract

Coronavirus disease 2019 (COVID-19) has swept the world, unlike any other pandemic in the last 50 years. Our understanding of the disease has evolved rapidly since the outbreak; disease prognosis is influenced mainly by multi-organ involvement. Acute respiratory distress syndrome, heart failure, renal failure, liver damage, shock and multi-organ failure are strongly associated with morbidity and mortality. The COVID-19 disease pathology is plausibly linked to the hyperinflammatory response of the body characterized by pathological cytokine levels. The term ‘cytokine storm syndrome’ is perhaps one of the critical hallmarks of COVID-19 disease severity. In this review, we highlight prominent cytokine families and their potential role in COVID-19, the type I and II interferons, tumour necrosis factor and members of the Interleukin family. We address various changes in cellular components of the immune response corroborating with changes in cytokine levels while discussing cytokine sources and biological functions. Finally, we discuss in brief potential therapies attempting to modulate the cytokine storm.

## 1. Introduction

In December 2019, several cases of pneumonia of unknown aetiology were observed in Wuhan, Hubei province, China. Soon after, the pneumonia was linked to the wet animal market in Wuhan, as the majority of patients that required medical attention had visited this market in the month previous to diagnosis. Reminiscent of the previous outbreaks, severe acute respiratory syndrome (SARS) in 2002 and Middle Eastern respiratory syndrome (MERS) in 2012, scientists immediately started isolating and identifying the pathogenic agent, a new member of the Coronaviridae family, later termed SARS-Cov-2. As of 12 March 2020, coronavirus disease 2019 (COVID-19) worldwide mortality was estimated at 3.7% [1]. This remains mostly unchanged. Moreover, it is estimated that 5% of the infected population will develop advanced diseases requiring intensive care, often necessitating extracorporeal organ support therapies. Of this critically ill subgroup, the mortality rate is high, at 40–50% [2].

In the majority of cases, individuals who test positive for SARS-Cov-2 by molecular diagnostics, typically reverse-transcriptase polymerase chain reaction (RT-PCR), may have no symptoms (termed asymptomatic or presymptomatic infection). On the other hand, COVID-19 symptomatology is typically associated with fever (98%), cough (76%), dyspnoea (55%) and myalgia or fatigue (44%). Other signs, such as sputum production (28%), headache (8%), haemoptysis (5%) and diarrhoea (3%), may also be present [3].

**Figure 1.**
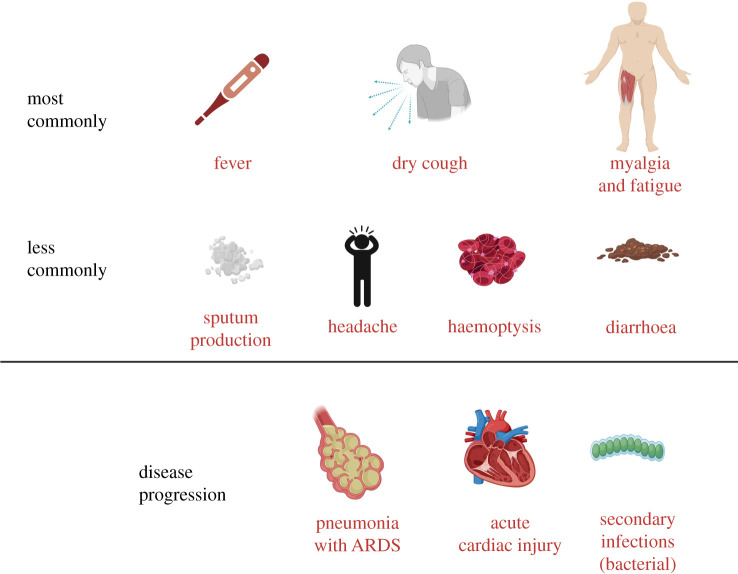
CS symptoms in COVID-19 and disease progression. Clinical symptoms of COVID-19 can be related to those associated with cytokine storm. Delayed detection of symptoms may lead to disease progression, with multi-organ involvement. It is difficult to manage and requires the admission of patients to ICU; intensive care is observed in about 5% of the infected population. Of the critically ill COVID-19 patients, the mortality rate is high, 40–50% [2,7].

In a clinical setting, severe disease is characterized by (infectious) pneumonia; complications typically include acute respiratory distress syndrome (ARDS) [4], acute cardiac injury [5] and secondary infections [6] (figure 1). ARDS is a significant complication in severe cases of COVID-19, affecting 20–41% of hospitalized patients [4,8], but heart failure, renal failure, liver damage, shock and multi-organ failure have also been observed in COVID-19.

Disease severity stratification depends on symptomatology [9]. Adult COVID-19 cases may be grouped as [10–13]:

1. Mild illness: individuals with any of the various signs and symptoms of COVID-19 (e.g. fever, cough, sore throat, malaise, headache, muscle pain) in the absence of shortness of breath, dyspnoea or abnormal chest imaging.
2. Moderate illness: individuals with signs of lower respiratory disease by clinical assessment or imaging and peripheral oxygen saturation (SpO_2_) ≥ 94% (room air at sea level).
3. Severe illness: characterized by breathing rates ≥ 30 breaths per minute, SpO_2_ < 94% (room air at sea level); a ratio of arterial partial pressure of oxygen to fraction of inspired oxygen (PaO_2_/FiO_2_) less than 300 mmHg, or lung infiltrates greater than 50%.
4. Critical illness: individuals presenting with respiratory failure (requiring mechanical ventilation), septic shock and/or multiple organ dysfunctions [9].

The multi-organ pathology is probably linked to the expression pattern of Angiotensin-converting enzyme 2 gene (ACE2). RNA expression is detectable across a wide range of human tissues [14]. The cells, tissues and organs most affected are those with high ACE2 expression, the entry receptor for SARS-Cov-2.

The extent of ACE2 protein expression detectable on the cell surface is still open to debate; previous work has shown that ACE2 is abundantly present in epithelia of the lung and small intestine in humans, opening the door for possible routes of entry for the SARS-Cov-2 [15]. However, more recent data suggest that cell-surface expression on the lungs is below the detection limit [16].

**Figure 2.**
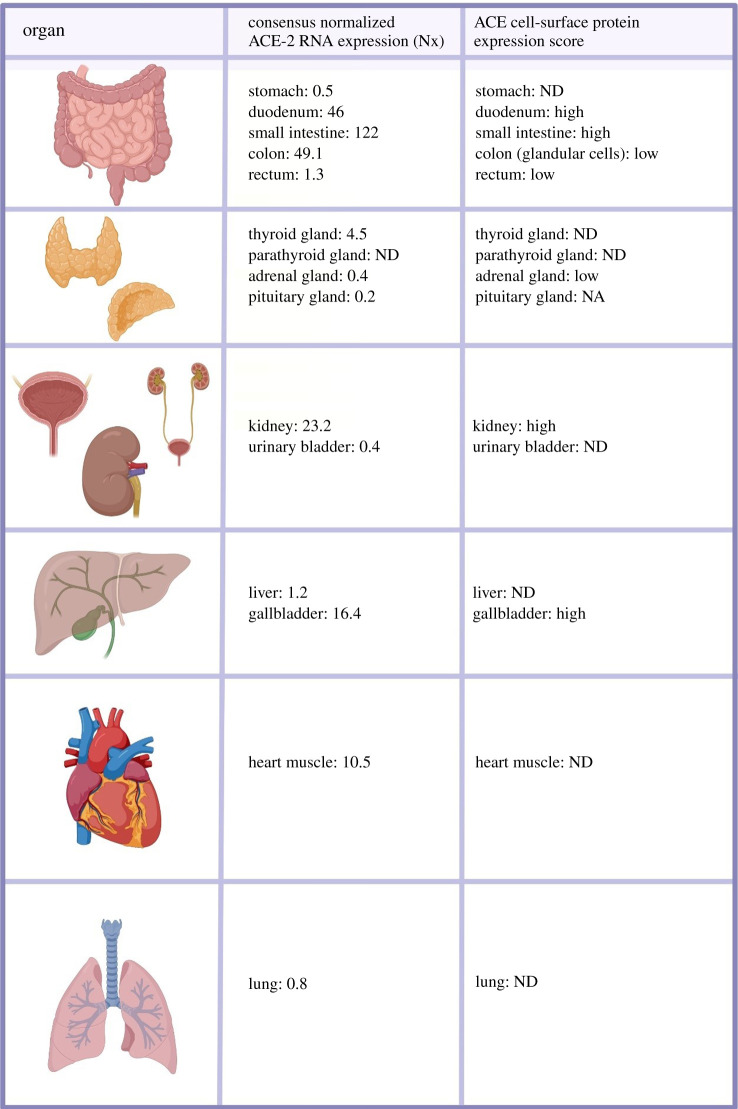
ACE2 expression in human tissues. SARS-Cov-2 uses the angiotensin-converting enzyme 2 (ACE2) as a cell receptor to invade human cells. ACE2 RNA and protein expression were observed in the endocrine tissues, gastrointestinal tract, the kidney and urinary bladder, the liver and gallbladder in men and women [7].

Considering the public data on the Human Protein Atlas, COVID-19 disease pathology does not correlate directly with ACE2 cell-surface protein expression (figure 2) [16]. However, the disparity may be associated with selective, transient expression of ACE2, as has been reported for the heart and kidneys [17,18].

## 2. Cytokine storm

Although the concept of an uncontrolled, cytokine-mediated response was already viable in the 1980s, first described in relation to malaria and sepsis [19,20], and subsequently in 2000s in the context of pancreatitis [21], variola virus [22] and influenza virus H5N1 [23], the first occurrence of the term ‘cytokine storm’ (CS) dates back to 1993 when it was reported in the context of graft-versus-host disease (GVHD) [24,25]. CS can be directly induced by a broad range of infections and by certain drugs. In the latter scenario, it is described as ‘infusion reaction’ or ‘cytokine release syndrome’.

CS is also a side effect of well-established medical practices like adoptive T-cell therapies (including CAR-T-cell therapy) [26] and the use of monoclonal antibody drug regimens [27,28] and immune checkpoint blockade inhibitors [29–31].

Mechanistically, a stressed or infected cell, through receptor−ligand interactions, activates large numbers of white blood cells, including B cells, T cells, natural killer cells, macrophages, dendritic cells and monocytes. This results in a release of inflammatory cytokines, which activate more white blood cells in a positive feedback loop. CS starts locally post-primary infection and spreads throughout the body via systemic circulation. The classical signs of inflammation—calour (heat), dolour (pain), rubor (redness), tumour (swelling or oedema) and loss of function—are observed. Initially, the localized response is meant to eliminate the trigger and involves protective mechanisms, i.e. increase in blood flow, facilitation of leucocyte extravasation and delivery of plasma proteins to the site of injury, increase in body temperature (advantageous in case of bacterial infections) and pain triggering (warns the host of the occurring challenge).

Nonetheless, repair processes are initiated soon after a pathogenic trigger. These processes can have two possible outcomes (1) organ function is gradually restored (2) healing occurs with fibrosis, which can result in persistent organ malfunction. Of note, the reported CS symptoms are not unique to SARS-Cov-2; similar observations were also made in SARS-Cov-1 and MERS-Cov cohorts [32,33].

Emerging data points to CS as a distinct immunological character of COVID-19; namely disrupted immune activation manifesting as hyperinflammation. Work by Ruan *et al*. [6] shows that the critically ill admitted to the ICU had higher systemic levels of IL-2, IL-7, IL-10, granulocyte-colony-stimulating factor, IP-10, monocyte chemoattractant protein-1 (MCP-1), macrophage inflammatory protein-1A (MIP-1A) and tumour necrosis factor-α (TNF-α) [6].

**Figure 3.**
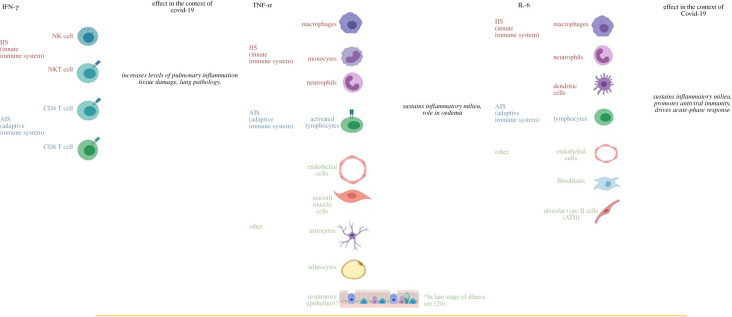
Prominent cytokine sources and their effect in COVID-19 pathogenesis. Immune cell sources of cytokines associated with cytokine storms; IFN-γ, TNF-α, IL-1 and IL-6 are depicted with their effects in the context of COVID-19 [7].

Importantly, data from recovered versus seriously ill patients, suggests that there is a significant association between severe uncontrolled inflammation and mortality. The main components of the CS are the critical immune elements of the pro-inflammatory milieu (figure 3).

In this review, we will be focusing on cytokines associated with ICU admission. These include the anti-viral cytokines of the interferon family, tumour necrosis factor and members of the interleukin family. Finally, we will elaborate on IL-6 and the current understanding of its involvement in light of the current COVID-19 pandemic.

## 3. Interferons (IFN)

IFN (type I, II, III) are central cytokines involved in innate immunity to bacteria and viruses. Type I and III interferons are produced, broadly, by all nucleated cells post-viral infection; interferon-α is predominantly produced by virus-infected leucocytes, and interferon-β by fibroblasts. Type II interferon (IFN-γ) is produced by macrophages, in response to viral and/or intracellular bacterial infections, and by natural killer (NK) cells. Additionally, IFN-γ is produced by T helper (TH) CD4 [34] and CD8 cytotoxic T lymphocyte (CTL) effector T cells during antigen-specific immunity [35]. Upon cognate receptor binding (IFNAR1/IFNAR2 for Type I interferons, IFN-γR1/IFN-γR2 for Type II interferons, receptor complex IL-28R/IL-10Rβ for type III—also known as lambda interferons), interferons activate a complex network of downstream signalling, which results in activation of transcription factors and induction of a multitude of IFN-stimulated genes that exert anti-viral, anti-proliferative or immunomodulatory properties.

Interestingly, lambda interferons (type III) have been reported to confer protection in a mouse model of influenza A virus [36]. In COVID-19, IFN-γ levels increased in corroboration with the viral load [3]. The delayed peak, paralleled with a drop in lymphocyte counts, increased neutrophil infiltration of the alveoli in the lungs, along with the deterioration of the patient's condition [3,37,38]. IFN-γ has been previously associated with disease severity. In SARS-Cov-1 and MERS-Cov, increased levels of IFN-γ was associated with pulmonary inflammation and extensive lung damage [39,40] both hallmarks of deterioration.

Along with IL-6, IFN-γ has been a reliable indicator of COVID-19 patient deterioration and ICU admission [37,38,41]. The source of IFN-γ has been up for debate, and it is widely accepted that CD4 TH cells are the direct source of IFN-γ, which promotes the differentiation of CD8 T cells and activates their cytotoxic abilities. CD4 TH cells produce granulocyte and monocyte colony-stimulating factor which promote monocyte differentiation (CD16^+^ CD14^+^ CD45^+^), which also sources IFN-γ in the blood.

## 4. Tumour necrosis factor-α

The tumour necrosis factor (TNF) superfamily consists of 19 members of type II transmembrane proteins that can be released from the cell membrane through extracellular proteolytic cleavage and function as cytokines. TNF-ɑ is produced by macrophages, monocytes, endothelial cells, neutrophils, smooth muscle cells, activated lymphocytes, astrocytes and adipocytes.

TNFR1 (primary receptor for TNF-α) is expressed by all cell types and thus responsible for the pleiotropic effects of this cytokine; TNF-α has a variety of functions, such as mediating the expression of genes for growth factors, cytokines, transcription factors and receptors. TNF-α plays a central role in CS. In COVID-19, TNF-α has been a prominent feature of patient deterioration, increasing in ICU patients in comparison to non-ICU [38,42].

Along with IL-6 and the soluble IL-2 receptor, TNF-α levels increase early in the infection and remain elevated throughout the infection [3,38]. Importantly, TNF-α in the lungs of COVID-19 patients induces HA-synthase-2 (HAS2) in EpCAM+ lung alveolar epithelium and CD31+ lung alveolar endothelium and fibroblasts [43]. HA (hyaluronan) is a key culprit for the fluid influx in the lung alveoli, a leading cause of deoxygenation and ventilator admission. Interestingly, lung epithelial cells do not secrete TNF-α in a model of highly pathogenic H5N1 influenza infection [44]; however, TNF-α expression by the lung epithelium is observed later as ARDS develops. TNF-α increase in expression is a result of macrophage-derived soluble factors interacting with lung epithelial cells [45]. In the circumstances of CS, this can be seen as a secondary effect to the ongoing pro-inflammatory cascade and highlights CS's ability to establish cross-talk with the affected mucosal tissue, to auto-sustain its amplification, resulting in the escalation of CS at a systemic level. It would be interesting to investigate whether SARS-Cov-2 infection is also able to drive TNF-α secretion by the lung epithelium as a result of CS.

## 5. Interleukins

Interleukins (ILs) regulate pro- and anti-inflammatory, immune cell differentiation and activation. Although initially thought to be exclusively involved in leucocyte-to-leucocyte communication (from which the term interleukin derived), they are now known to be produced by a wide variety of cell types.

IL-1 plays an essential role in T-cell-derived immunity, promoting IL-2 secretion, a key player in T-cell homeostasis [46], as well as IL-2 receptor expression [47,48]. IL-1α and IL-1β increase acute-phase signalling, trafficking of immune cells to the site of primary infection, epithelial cell activation, and secondary cytokine production. The acute-phase response to infection is evidenced in a wide range of local and systemic effects that are generally pro-inflammatory, such as the increase in specific cytokine production, which can be linked to viral clearance. IL-1α expresses costimulatory function strictly on TH2 cells, with little to no effect on TH1 cells [48]. The IL-1 high-affinity receptor, IL-1RI, is highly expressed on TH2 cells [49]. In models of hypersensitivity, IL-1α/β−/− mice had lower IL-4 and IL-5 levels compared to controls, reducing symptoms of allergy [50].

By contrast, IL-1α/β proved critical in sustaining a TH2 immune milieu in a murine *trichuris muris* infection, necessary to overcome the parasitic infection [51]. Additionally, IL-1 plays important roles in TH17 induction and functionality. IL-1RI−/− mice mounted less TH17 cells compared to wild-type controls [52]. Interestingly, IL1RI−/− mice were resistant to experimental autoimmune encephalomyelitis [52]. Of note, the induction of TH17 in autoimmune experimental models requires IL-1β (induced artificially by killed inactive *Mycobacterium tuberculosis*) [53]. In the respiratory tract, IL-1 receptor signalling is responsible for acute lung immunopathology and enhancing the survival of mice infected with influenza virus by inducing IgM antibody responses and recruiting CD4 T cells to the site of infection [54].

In COVID-19, patient CT lung scan with multiple bilateral lobular pneumonia is associated with IL-1β, IL-7, IL-8, IL-9 level increase in initial plasma concentration [3]. These cytokines are released from damaged tissue and are early immune drivers of the immune response in COVID-19. Strikingly, this increase was similar in both ICU and non-ICU patients, suggesting their profound involvement in the immunopathology of COVID-19 [3]. Moreover, it was observed that IL-2 and IL-7 increased in ICU and non-ICU patients [3,55]. Similarly, IL-10 increases to a similar pattern to IL-2 and IL-7 [3]. IL-10 is understood to be released form antigen-presenting cells responsible for the differentiation and activation of CD8 T cells and TH cells as a feedback response to the increased levels of IFN-γ and IL-6. It appears that IL-10, a potent immune modulator, in the case of COVID-19, is considered a vital sign of immune delinquency. IL-10 levels are increased in the second week following symptom-onset, while not associated with the immune drawback, it is an indication of latent immune efforts to control the CS [38], which are unfortunately too late. IL-4, a TH2 cytokine and suppressor of inflammation, also, increases in ICU patients in a late regulatory attempt by the immune system [3]. Collectively, ILs, while not archetypical anti-viral cytokines like IFNs, however, no doubt shape CS morbidity.

## 6. IL6: in the eye of the storm

Human IL-6 is made up of 212 amino acids, including a 28-amino acid signal peptide, and its gene has been mapped to chromosome 7p21. Although the core protein is 20 kDa, glycosylation accounts for the size of 21–26 kDa of natural IL-6. In the immune system, IL-6 plays many essential roles that help shape anti-viral immunity. IL-6 is a prominent pro-inflammatory cytokine with a range of inflammatory roles. Interleukin 6 (IL-6) is an interleukin that affects the activity of a variety of cell types. Hence it is described as a pleiotropic cytokine and acts both as a pro-inflammatory cytokine and an anti-inflammatory myokine (a specific type of cytokine expressed by muscle cells in response to muscular contraction).

Upon its production, IL-6 binds to its soluble receptor and forming the IL6/IL6R complex. IL-6 binds to its receptor, which is expressed on a broad range of immune cells and tissues. The IL-6 receptor-signalling complex comprises of two transmembrane-IL-6 binding chains, two soluble IL6 receptors, and two cytoplasmic signalling molecules (gp130). The IL-6R cytoplasmic signalling molecule is shared by other members of the IL-6 family, i.e. leukaemia inhibitory factor, IL-22, IL-27 and IL-25.

Consequently, the receptor co-sharing possibly forms the basis for the collective redundancies and pleiotropic effects shared between IL-22, IL-27 and IL-25 and functions attributed to IL-6. The binding of soluble IL-6 to its ligand upregulates the gp130. The binding allows for the formation of the IL-6/IL-6R complex, which in turn triggers the downstream signalling of the IL-6-related intracellular cascade. The intracellular cascade following complex formation involves the downstream activation of the Janus kinase (JAK)-STAT3 pathway and the JAK-SHP-2-MAP kinase pathway. STAT3 regulates IL-6 responses by inducing suppressor cytokine signalling-1 (SOCS1) and SOSC3, which negatively oppress IL-6 signalling inhibiting intracellular feedback loops.

A wide range of immune cells secrete IL-6, i.e. macrophages, neutrophils, dendritic cells and lymphocytes. Importantly, the release of IL-6 within an inflammatory milieu is due to the vast number of cells that secrete it that are structural components of the infected tissue and not necessarily part of the immune system *per se*, i.e. mesenchymal cells, endothelial cells, fibroblasts and others are involved in the production of IL-6. These findings highlight the abundance and profound potential IL-6 carries in an inflammatory condition. Functionally IL-6 arrives, through the bloodstream, at the liver and rapidly activates hepatocytes to produce C-reactive proteins, serum amyloid A and promotes the release of fibrinogen.

Moreover, hyperinflammation may be accompanied by a drop in albumin, which is an indication of liver damage and, more importantly, systemic disease. Centrally, IL-6 promotes the differentiation of naïve CD4 T cells into effector and helper cells [56]. As it bridges natural immunity into adaptive immune responses, IL-6 promotes TH7 differentiation [57] along with cytotoxic CD8 T lymphocytes activation and differentiation [58].

Additionally, IL-6 inhibits the production of T regulatory T CD4^+^CD25^+^ FOXP3 cells [59], therefore attributing to the development of a long list of autoimmune diseases. Immunoglobulin production is regulated indirectly by IL-6 through promoting T-follicular helper cell, B cell and plasma cell differentiation as well as IL-21. On a side note, some viruses can manipulate the intracellular cascade of events attributing to the inflammatory status and the release of IL-6. An example of this is HIV-1, which enhances the DNA binding activity of NFkB and NF-IL-6, increasing IL-6 RNA transcription and as a subsequent effect excessive IL-6 secretion. A similar mechanism has been shown for SARS-Cov-1: specifically, the SARS-Cov-1 structural protein N (nucleocapsid), but not protein S (spike), protein E (envelop) and protein M (membrane) significantly induced the activation of IL-6 promoter in human airway epithelial cell cultures, via direct binding of NFkB regulatory element on IL-6 promoter [60] This could, in theory, counteract IL-6 regulatory mechanisms that lead naturally to the cessation of IL-6 mediated activation once the threat is resolved. The mechanisms would need further investigation to assess potential long-term effects of COVID-19, especially considering the environmental factors and the development of autoimmune disorders, i.e. transient autoimmune disorders that follow viral infections.

IL-6 has been in the centre of this COVID-19 pandemic [61]. Early in the outbreak, IL-6 levels were a reliable indicator of disease severity and predictive in terms of ventilation support [6,62,63]. Pedersen and colleagues discuss that increased levels of IL-6 (along with TNF-α and IL10) is significantly associated with reduced levels of recovery chances and requiring ICU admission [38]. Additionally, in their quantitative study, mild to moderate levels of IL-6 and others corroborated with moderate cases. Prompetchara and colleagues identified a 52% increase in the level of IL-6 in ICU patients compared to non-ICU [41]. This was associated with neutrophilia and lymphocytopenia and an increase in CRP levels. Zhao and colleagues showed that both IL-6 and IFN-γ are indirectly promoted by CD4 TH lymphocytes through the secretion of GM-CSF [64]. GM-CS, in turn, induces the production and recruitment of CD14+CD16+ monocytes that release IL-6 into the pulmonary environment.

As observed in SARS-Cov-1 and MERS, IL-6 is generated early in the infection as a result of innate, MyD88-dependent pathway, immune receptor activation following the detection of viral proteins inside the infected cell. Notably, evidence for IL-6 production enhancement in SARS-Cov-1 pathogenesis [60] builds the ground for the hypothesis that the two members of the Coronaviridae family might indeed share common physiopathological mechanisms and points out to new possible strategies for therapeutic interventions [65].

## 7. Antigen-independent, cytokine-dependent amplification of the inflammatory loop

Viral antigens are typically the initial trigger of innate and adaptive immune activation [66]. Screening of circulating T cells using HLA class I and II predicted peptide ‘mega pools’ identified SARS-Cov-2-specific CD4 and CD8 and T cells in approximately 100% and 70% of COVID-19 convalescent patients. The most robust CD4 T-cell responses targeted the viral Spike protein, and these responses correlated with anti-SARS-Cov-2 IgG and IgA levels across the cohort.

CD8 T-cell responses were also predominantly directed to the spike protein. The second most dominant antigen identified was the M (membrane) protein [66]. This data justifies the ongoing vaccine efforts directed at the SARS-CoV-2 Spike protein [67].

T-cell receptor (TCR) recognition of HLA/peptide–epitope complexes triggers activation and differentiation of naive T cells; as a result, T cells acquire distinct phenotypic and functional properties as well as effector functions [68–70]. A model of T-cell responses in COVID-19 suggests that disease severity may be associated with early cytokine programming of naive T cells [71]. Mild disease is associated with IL-2, type I and type III interferon. Conversely, severe disease is linked to IL-6, IL-10, IL-1­, TNF and CXCL8 and other CXCLs during T-cell priming.

Undoubtedly, viral persistence and continuous antigen exposure determine clinical course [72]. Viral load also correlates with pro-inflammatory cytokine levels [73,74].

However, there are also reports pointing to a plausible antigen-independent, cytokine-dependent immune amplification sustaining hyperinflammation in COVID-19. For instance, the early case series by Lescure *et al.* reporting late respiratory deterioration despite the disappearance of nasopharyngeal viral RNA suggests that late, severe manifestations may be (mainly) immunologically mediated [75].

With the discovery of cross-reactive Cov memory T cells in healthy donors [66], one should consider that memory T cells might be involved in the COVID-19 pathogenesis. The common-γ-chain cytokines play a major role in health and disease [76]; IL-7, IL-15 are known drivers of antigen-independent, homeostatic, memory T-cell proliferation and bystander T-cell proliferation [77–80]. Moreover, IL-2 secreted by activated T cells may also promote bystander activation [79–81]. Lucas *et al*. showed that IL-7, IL-15 and IL-2 were increased in COVID-19 and correlate with disease severity [73] and may promote IFN-γ production in an antigen-independent manner [82].

Other cytokines may also be involved in potentiating the inflammatory loop; naive and memory virus-specific CD8 T-cell activation can be achieved in an antigen-independent manner with cytokine cocktails, e.g. IL-12 + IL-18 [83]. These cytokines may trigger rapid antigen non-specific IFN-γ secretion during infections with intracellular pathogens [84].

Provided that several cytokines are increased during COVID-19, it is important to underline the synergistic potential of cytokine ‘cocktails’. For example, subthreshold amounts of TNF-α with IL-12 leads to greater than 20-fold increase in IFN-γ producing CD8 T cells in comparison to IL-12 mono-stimulation [83]. In a dengue virus model, IFN-γ production from CD4 and CD8 T cells in a TCR-independent manner involving IL-12 was reported [85]. During chronic viral infections, prolonged Antigen exposure has been shown to generate innate-like CD8 T cells that respond to cytokines in the absence of TCR-stimulation [83].

Increased levels of IL-1β in COVID-19 may point to inflammasome activation [86,87]. However, it has also been shown that cognate interactions between effector CD4 T cells and myeloid cells via the TNF/TNFr axis can trigger IL-1β production [88]. IL-1β selectively expands and sustains IL-22 producing immature natural killer cells, and IL-22 is linked to Type 3 immune responses during infections of extracellular bacteria and fungi. Interestingly, severe COVID-19 patients show hallmarks of heightened type 3 responses, including increased levels of IL-17 and IL-22 [73].

In summary, cytokines may support antigen-specific and antigen-independent immune activation. In the absence of regulatory mechanisms, cytokine amplification may convert to cytokine storm.

## 8. Discussion

COVID-19 disease presentation resembles clinical observations reported for the hyperinflammatory syndrome ‘secondary haemophagocytic lymphohistiocytosis' (HLH) [89].

Some have postulated that COVID-19 should be included within the broader spectrum of hyperinflammatory syndromes [90]. Clinically, HLH is associated with hepatomegaly and splenomegaly. Probability for HLH in COVID-19 may be estimated using a risk algorithm in line with established diagnostic criteria [91–93]. Assessing COVID-19 from the biochemical perspective the disease has also been linked to hypertriglyceridaemia [94], another overlapping parameter with hyperinflammatory syndromes such as HLH.

One more similarity with HLH is hyperferritinemia; severe COVID-19 cases are characterized by significantly higher ferritin levels [6]. Ferritin biology during infection is beyond the scope of this review, but for an in-depth review we kindly refer the readers to the article by Kernan *et al.* [95].

The aforementioned clinical manifestations are strongly linked to the uncontrolled immune response observed in COVID-19.

COVID-19 has been shown to elicit a two-phase immune response; in the initial (asymptomatic, pre-incubation) phase the adaptive immune response plays a critical role in its attempt to kill infected epithelial cells and thereby by preventing viral replication [43]. The second phase points to a failure of the adaptive immunity to clear the virus; consequently, SARS-Cov-2 propagates.

Subsequent viral budding is associated with NACHT, LRR and PYD domains-containing protein 3 (NLRP3) inflammasome activation [86] and immunogenic cell death [87].

Recently, Zhou *et al*. [96] and Hoffmann *et al*. [97] collectively elucidated the mechanisms of cell entry employed by SARS-Cov-2. Both reports showed that SARS-Cov-2 engages ACE2 as the entry receptor and, importantly, requires the binding of the viral spike (S) proteins to cellular receptors in conjunction with S protein priming by the serine protease TMPRSS2 [97].

The importance of TMPRSS2 is highlighted as the authors showed that a clinically approved TMPRSS2 inhibitor could effectively inhibit viral entry, opening the door for expedited clinical testing in COVID-19 patients [98].

**Figure 4.**
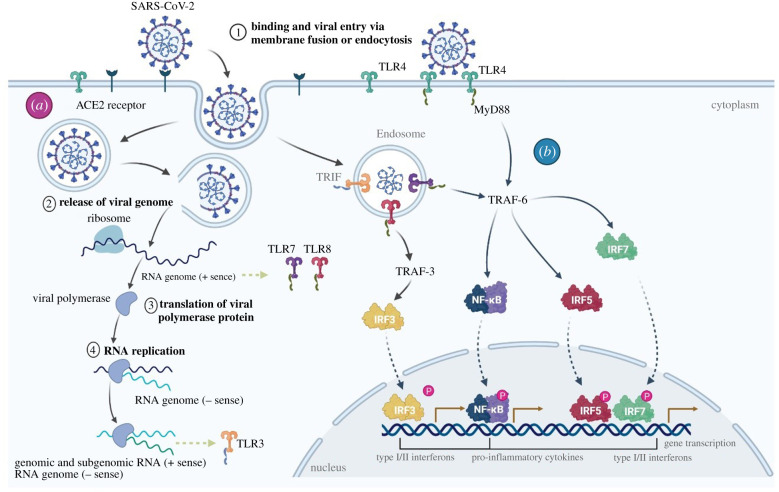
Viral entry, replication and innate immune activation. Multiple distinct toll-like-receptors (TLRs) pathways are involved in SARS-CoV-2 pathogenesis. SARS-Cov-2 cellular entry and subsequent replication (*a*) may trigger the immune system by engaging multiple TLRs (*b*). The spike protein triggers TLR4, ssRNA activates TLR7 and dsRNA may lead to TLR3 activation. Following TLR activation, IRFs and NF-*κ*B-dependent signalling pathways are activated leading to type I/II Interferons and pro-inflammatory cytokines [7].

Viral entry and replication may trigger distinct Toll-like receptors (TLR) and downstream signalling pathways (figure 4). The importance of TLR-sensing to elicit a robust anti-viral immunity has been thoroughly studied [99–101]. In particular, TLR7 was shown to be imperative to control COVID-19 disease severity; Van der Made *et al*. showed that loss-of-function variants of the X-chromosomal TLR7 result in diminished type I and II IFN responses linked to among others to decreased mRNA expression of IRF7 (figure 4).

Given the prominent role of TLR7, Imiquimod, a TLR7 agonist has been proposed as an option to boost anti-viral immunity [102]. Targeted-nanoparticle vaccines [103] may be explored for effective *in vivo* delivery to dendritic cells and to elicit a robust adaptive immune response [104].

The apoptotic cascade and massive destruction of infected tissues trigger an innate-like inflammation [100,105,106]. IL-6 is generated early in the infection as a result of innate, MyD88-dependent pathway, immune receptor activation by endogenous viral proteins [107]. SARS-Cov-2 infection in the respiratory system was also shown to activate the IL-6 amplifier (IL-6 Amp) in an NF*κ*B and STAT3-dependent manner. IL-6 amplification may contribute to the hyperinflammation observed in COVID-19 similar as seen in multiple inflammatory and autoimmune diseases [61,108].

**Figure 5.**
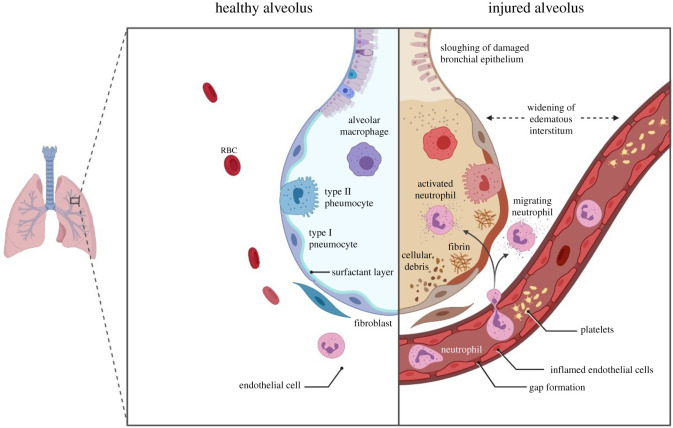
ARDS is acute condition occurring within 1 week of clinical insult, or the onset of respiratory symptoms, characterized by bilateral pulmonary immune infiltrates and severe hypoxaemia in the absence of cardiac failure or pulmonary edema. The condition is characterized by severity levels based on the PaO2/FiO2 ratio; mild (PaO2/FiO2 200–300), moderate (PaO2/FiO2 100–200), and severe (PaO2/FiO2 ≤ 100) [7].

The local inflammatory milieu attracts a diversity of immune cells [109], activated CD4 T cells [66], monocytes and macrophages [110] may further stimulate IL-6 Amp leading to a pathological positive feedback loop. Thus, initial secretion of IL-6 by infected epithelial cells sets the stage for massive infiltration by activated pro-inflammatory immune cells which further increase the local cytokine levels and catalyse the adverse inflammatory milieu; particularly in the lungs, the ensuing lung inflammation is the leading cause of life-threatening ARDS (figure 5) at the severe stage.

The association between lung pathology and COVID-19 is well established; however, recent reports are pointing to the involvement of other organ dysfunction, among others, acute kidney injury (AKI) [111,112]. Nevertheless, our understanding of the mechanisms and the distinct pathophysiology of COVID-19 induced AKI is still in its infancy [113,114].

Numerous studies have implicated a role for IL-6/IL-6r in the pathophysiology of AKI [115–117]. Furthermore, levels of IL-6 in kidney injury patients strongly correlate with mortality in a concentration-dependent manner [118].

Several mechanisms have been proposed to clarify the role of IL-6 in renal impairment; for instance, IL-6 can promote renal disease by increasing sensitivity of tubular epithelial cells to pro-fibrotic cytokines such as TGF-β. Also, it has been shown that IL-6 worsens mesangial proliferative glomerulonephritis by inducing mesangial cell proliferation [117,119].

Furthermore, hyperinflammation has been associated with COVID-19 induced coagulopathy [120,121], a result of increased production of clotting factors by the liver under continuous cytokine stimulation [122]. Indeed, post-mortem reports describe signs of thromboembolism [123].

We [124] and others [125] have observed that hospitalized COVID-19 patients show extremely elevated D-dimers (greater than 500 ng ml^−1^) and Fibrinogen (greater than 5.5 g l^−1^) levels; some severely ill patients are admitted with D-dimers of greater than 20 000 ng ml^−1^ evidencing a severe hypercoagulable state.

## 9. COVID-19 for avenues therapeutic

Currently, there are no FDA-approved therapies for the treatment of COVID-19 [126]. Nonetheless, numerous studies and observations have pointed to a potential clinical benefit of controlling hyperinflammation triggered by SARS-Cov-2 as seen frequently in COVID-19 cases [6] as a means to halt disease progression.

Still, the current management of COVID-19 is mostly supportive and based primarily on continuous respiratory support. Considering the cytokine data and clinical observations pointing to an underlying immunological character of COVID-19, we suggest further studies in the area of immune modifying therapeutics. Indeed, immunomodulators are the biggest group of therapeutics undergoing accelerated testing [126].

Given the convincing role of IL-6 in COVID-19 pathology, neutralization of the IL-6/IL-6r via Tocilizumab (a recombinant humanized anti-IL-6 receptor (IL-6r) monoclonal antibody (mAb), Sarilumab (a recombinant humanized anti-IL6r) and Siltuximab (a recombinant human-mouse chimeric monoclonal antibody that binds IL-6) may attenuate CS [127,128] and also prevent renal function impairment [129].

Another therapeutic intervention pertains to IL-1 blockade with Anakinra. The IL-1r antagonist is a cornerstone treatment for hyperinflammatory conditions—its use has a favourable safety profile event at high dosages and hence suggested a therapeutic approach in COVID-19 [130].

Anakinra is administered to inhibit the pathological effects of IL-1α and IL-1β. Two cohort studies have evaluated the clinical effectiveness with promising results [131–133]. In the absence of randomized trials, FDA recommends that clinicians consider their use with caution [134].

Blocking systemic inflammation by targeting specific cytokines through antibody-mediated neutralization have so far yielded mixed results in clinical settings [135–138] or effectiveness in selected subgroups.

Consequently, considerable research efforts have focused on alternative strategies aimed at non-specific sequestration of inflammatory mediators; for example, blood purification through filtration, dialysis (diffusion) and adsorption [139–142].

The overall concept of blood purification is to attenuate the pathogenic systemic levels of pro-inflammatory mediators. Restoration of immune homeostasis, namely decreased IL-6 levels [116], is thought to be able to decrease the incidence of COVID-19 induced AKI and thus improves outcomes and survival.

Several recent reports provide promising observations in regards to the control of hyperinflammation; cytokine adsorption, blood purification, effectively decreased levels of IL-6 in advanced COVID-19 disease [124,130,143]. Nevertheless, randomized controlled trials are warranted to determine the calibre of blood purification regimens to promote clinical recovery of COVID-19 patients.

## 10. Summary

In this review, we highlight the most identifiable targets within the fatal cytokine response in severe COVID-19 patients that have been identified via rigorous studying of the current, and vastly expanding, research on the topic. In the time of writing this review, many therapeutic drugs are undergoing clinical testing; Unsurprisingly, IL-6 blockade is currently the main target [144–146]. However, TNF-α blockade should also be explored [147,148].

Clinical trials, while in their infancy, carry enormous hope for ending the suffering of COVID-19 patients.

## Data accessibility

This article has no additional data.
